# Treatment of Bilateral Giant Fusiform Petrocavernous Aneurysms

**DOI:** 10.7759/cureus.8662

**Published:** 2020-06-17

**Authors:** Koushik Mantripragada, Nikolas Echeverry, Samuel Mansour, Eric C Peterson, Brian Snelling

**Affiliations:** 1 Medicine, Charles E. Schmidt College of Medicine at Florida Atlantic University, Boca Raton, USA; 2 Neurosurgery, Charles E. Schmidt College of Medicine at Florida Atlantic University, Boca Raton, USA; 3 Neurosurgery, University of Miami Miller School of Medicine, Miami, USA; 4 Neurosurgery, Boca Raton Regional Hospital, Boca Raton, USA

**Keywords:** petrocavernous aneurysm, treatment of petrocavernous aneurysm, bilateral giant fusiform aneurysm

## Abstract

Aneurysms of the petrous segment of the internal carotid artery (ICA) are exceedingly rare. They are thought to arise from traumatic, mycotic, or congenital etiologies. We present a case of bilateral giant fusiform aneurysms of the petrocavernous ICA treated with bilateral flow-diverting stent placement.

An 18-year-old male presented to our institution with headaches, nausea, vomiting and blurry vision that had been present since the day prior. Visual exam revealed decreased visual acuity bilaterally and a temporal field cut superiorly and inferiorly of the left eye. CT and MR imaging revealed bilateral lesions of the petrous segment of the ICA bilaterally. Catheter angiography demonstrated bilateral giant fusiform aneurysm of the petrocavernous ICA. The patient was treated with aspirin 325 mg and clopidogrel 75 mg orally daily for one week prior to the exam. VerifyNow (Accriva; San Diego, CA) confirmed adequate platelet inhibition. The right ICA was treated first, with a multiple flow-diverting stent construct. No complications were noted and the patient was discharged to home two days later. He was brought back three weeks later, and the left ICA was treated with a multiple flow-diverting stent construct. Again, no complications were noted and the patient was discharged uneventfully. The patient returned for his six-month follow-up angiogram with improvement of his visual acuity and resolution of headaches. However, the patient had ceased taking both anti-platelet medications six weeks prior. Angiography revealed no filling of the aneurysm in the right ICA, however, the left ICA was occluded at the origin. The patient was resumed on daily aspirin 325 mg orally and will have follow-up catheter angiography at 12 months.

Petrous segment ICA aneurysms are rare. Most are thought to arise from trauma, infection, or congenital etiologies. These aneurysms are typically fusiform in nature, and can extend into the cavernous segment of the ICA. The natural history of these aneurysms is not well understood given their rarity. Current literature advocates for asymptomatic patients to be treated conservatively given that the natural history is not well known. Treatment is recommended in symptomatic patients, who may present with symptoms of local mass effect or ischemic stroke due to emboli. Endovascular options include flow diverting stent or covered stent placement, coil embolization with or without stent-assistance, or ICA occlusion. Open surgical options include trapping and high-flow bypass.

When bilateral lesions are present, the management algorithm must be amended. We elected to treat the asymptomatic side first (right ICA), due to the presence of a significant kink within the aneurysm on the left. Once the right side was treated successfully, the symptomatic side was treated with a multiple stent construct.

The patient’s six-month angiogram demonstrated occlusion of the left ICA, likely due to non-compliance with antiplatelet medications. This further reiterates the need for dual-antiplatelet therapy and patient education and compliance with flow diverting stents.

We report a rare case of bilateral giant fusiform petrocavernous aneurysms treated with bilateral Pipeline embolization devices in multiple device construct, demonstrating the feasibility and safety of this treatment option for this pathology.

## Introduction

Aneurysms of the petrous segment of the internal carotid artery (ICA) are exceedingly rare [1,2]. Treatment of aneurysms within the petrous ICA is further complicated by the architecture of this portion of the carotid artery. The petrous segment begins where the ICA penetrates the temporal bone at a 90-degree angle from which it initially ascends vertically (vertical segment, ~10 mm) until turning anteromedially (genu segment) to become the horizontal segment where it continues for ~20 mm towards the petrous apex, finally ending at the foramen lacerum [3]. Aneurysms that form within this segment of the ICA are enclosed by the temporal bone and thus more difficult to treat than those within other segments of the ICA that are not enclosed within the skull base [4].

These aneurysms can be further subclassified into true aneurysms and pseudoaneurysms of the petrous ICA. True aneurysms are thought to arise from trauma. Pseudoaneurysms do not have a true wall and arise when a thrombus and fibrous tissue capsule develops possibly secondary to blunt or penetrating trauma, infections, inflammation, or radiation. Other possible causes of aneurysm development include mycotic origins or congenital etiologies. Aneurysm development from mycotic origins is likely secondary to an adventitial infection, such as chronic otomastoiditis, tonsillar and pharyngeal infections, and even cholesteatomas [1]. Aneurysm development from congenital origins likely arises from developmental defects in the muscular middle layer of the vessel.

We present a case of bilateral giant fusiform aneurysms of the petrocavernous ICA treated with bilateral flow-diverting stent placement. Aneurysms of the petrous segment of the ICA are rare, and bilateral giant fusiform aneurysms are exceedingly rare.

## Case presentation

An 18-year-old male presented to our institution with headaches, nausea, vomiting and blurry vision that had been present since the day prior. Visual exam revealed decreased visual acuity bilaterally and a temporal field cut superiorly and inferiorly of the left eye.

**Figure 1:**
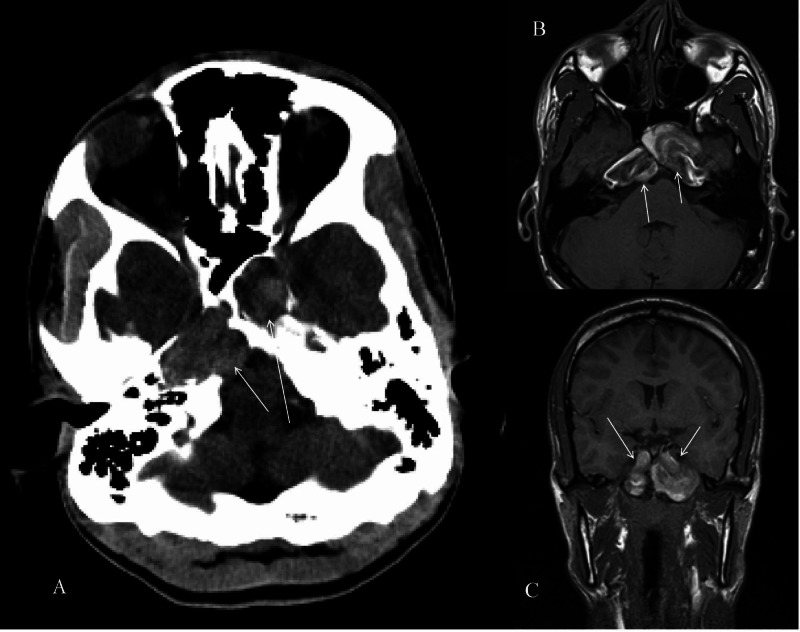
CT transverse (A), MRI transverse (B), and MRI coronal (C) revealed bilateral lesions of the petrous segment of the internal carotid artery (ICA) bilaterally

**Figure 2:**
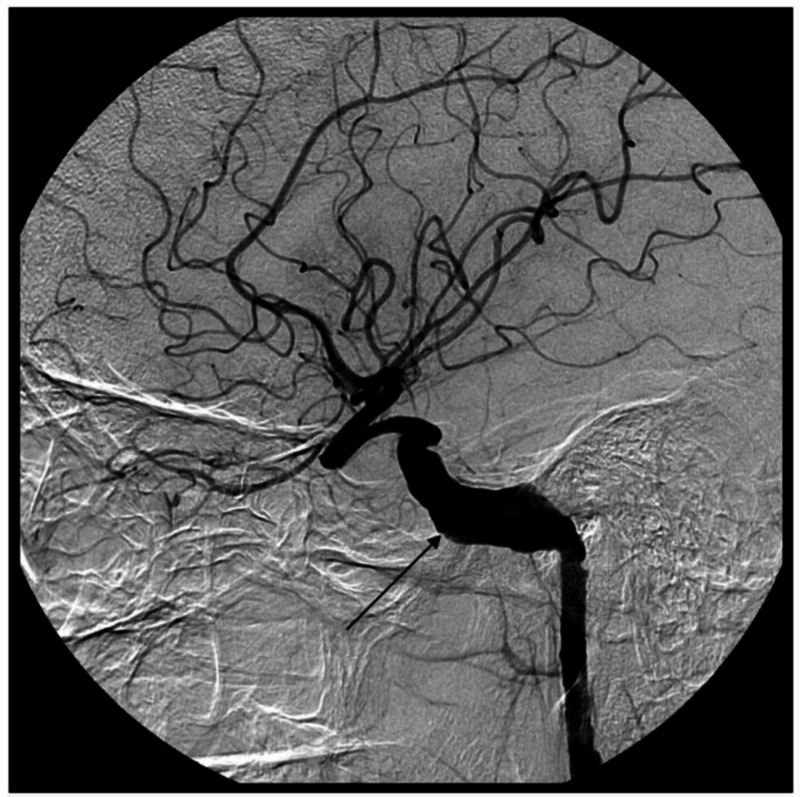
Catheter angiography demonstrated bilateral giant fusiform aneurysm of the petrocavernous internal carotid artery (ICA)

CT and MRI revealed bilateral giant fusiform aneurysms of the petrous portion of the internal carotid artery (Figure 1), which was confirmed with catheter cerebral angiography (Figure 2).

The patient was started on aspirin 325 mg and clopidogrel 75 mg to achieve adequate platelet inhibition. VerifyNow (Accriva; San Diego, CA) confirmed adequate platelet inhibition.

The left ICA aneurysm had a significant kink that would present a challenge for reconstruction with flow diversion. If the left ICA were not able to be reconstructed successfully and subsequently occluded, the patient would have depended on the diseased right ICA for supplying both hemispheres. Thus, the right ICA aneurysm, though asymptomatic, was treated first to ensure adequate treatment prior to treatment of the left ICA. The right ICA aneurysm was treated using a multiple-flow diverting stent construct with the Pipeline Embolization Device (Medtronic, Irvine, CA), followed by the left ICA aneurysm three weeks later. He was brought back three weeks later, and the left ICA was treated with a multiple flow-diverting stent construct.

After each individual treatment, there were no complications noted, and the patient was discharged. The patient returned for his six-month follow-up angiogram with improvement of his visual acuity and resolution of headaches. However, the patient had ceased taking both anti-platelet medications six weeks prior. Angiography revealed no filling of the aneurysm in the right ICA, however, the left ICA was occluded at the origin. The patient was resumed on daily aspirin 325 mg orally and will have follow-up catheter angiography in six months.

## Discussion

Despite being considered intracranial aneurysms, petrous aneurysms in asymptomatic patients are managed conservatively because they are extradural [1,2]. Patients with asymptomatic extradural ICA aneurysms are typically managed with long-term observation and management of controllable risk factors such as high blood pressure and tobacco use [4-6].

Depending on their size and location, some petrous aneurysm may cause symptoms including headaches, tinnitus, hearing loss, visual changes, or other symptoms resulting from mass effect [1,4,7]. For those patients with either symptomatic presentation or documented growth, neurosurgical intervention is recommended [2]. If the patient is asymptomatic, watchful observation is encouraged given the limited data regarding the natural history of these lesions.

Interventions typically utilized in the treatment of unruptured intracranial aneurysms include ICA occlusion (with or without high flow external carotid to internal carotid bypass), aneurysm embolization, and flow diverting stent placement [5]. The Pipeline embolization device was utilized because it provides permanent aneurysm occlusion without temporary or permanent occlusion of the parent vessel [8,9].

In the presented case, we utilized the Pipeline embolization device, a type of flow diverting stent. The Pipeline embolization device is a self-expanding endoluminal mesh tube which diverts blood flow within the ICA past the site of aneurysm, allowing for uninterrupted blood flow while simultaneously leading to thrombosis of the target aneurysm [10]. Within the past decade, flow diverters have become a commonly utilized intervention in the management of intracranial aneurysms [9]. Flow diverting stents offer several advantages over traditional interventions including complete and permanent aneurysm occlusion as well as providing scaffolding over which a neointimal layer can grow at the diseased portion of the ICA [9]. Additionally, for certain aneurysms, they provide decreased risk of procedural complications which are more commonly seen in open aneurysmal clipping while also reducing the aneurysm recurrence seen in endovascular coiling [8,9].

## Conclusions

Petrous aneurysms are already uncommon; bilateral petrous aneurysms are even more so, thus there is no defined treatment algorithm for the management of these lesions. Given the patient's left temporal hemianopsia as well as the increased complexity of the left ICA anatomy, the right-sided aneurysm was treated with the flow diversion first to preserve arterial supply to both hemispheres in the event the left ICA could not be reconstructed successfully. At the six-month follow-up, the patient reported non-compliance with antiplatelet medications and angiography showed occlusion of the left ICA. In spite of this, the right ICA demonstrated normal flow with no filling of the right ICA aneurysm. Given that no procedural complications were noted and the asymptomatic left ICA occlusion was likely due to medication noncompliance, we can conclude that this treatment option is both safe and efficacious in the management of bilateral petrous aneurysms.
